# Supplementary material for: Ontogeny of the Cytochrome P450 Superfamily in the Ornate Spiny Lobster (Panulirus ornatus)
Source: Int J Mol Sci. 2024 Jan 15;25(2):1070. doi: 10.3390/ijms25021070 (PMC10816631; doi:10.3390/ijms25021070)
Supplement: Supplementary file 1 [file ijms-25-01070-s001.zip › ijms-2788952-supplementary.pdf]

Table S1. CYP450 expression across the 52 tissue libraries of juvenile and adult *Panulirus ornatus*. Tissues were collected from 1 male (first box of each tissue) and 1 female (second box of each tissue) except for testes (3 juveniles, 3 adults), sperm duct (3 regions from one adult male) ovaries (3 juveniles, 3 adults), oviduct (one adult female), hepatopancreas (2 juvenile males, 3 adult males, 3 juvenile females, 3 adult males). Transcripts ordered according to expression similarities across tissues. ES (eye stalk), Br (brain), TG (thoracic ganglia), Ant (antennal gland), Ts (testes), SD (sperm duct), Ov (ovary), M Hp (male hepatopancreas), F Hp (female hepatopancreas), G (gill), He (heart), Md (midgut), Hd (hindgut), Ms (muscle), Ep (epidermis), Ft (Fat), H (hemolymph). Shades of blue indicate expression levels, ranging from light blue (low expression) to the darkest blue (highest expression), represented as RPKM.

	ES	Br	TG	Ant	Ts	SD	Ov	M Hp	F Hp	G	He	Md	Hd	Ms	Ep	Ft	H
Cluster-70816.42385																	
Cluster-70816.135374																	
Cluster-70816.50435																	
Cluster-70816.72997																	
CYP3A2-like_Pan_orn																	
Cluster-70816.139448																	
CYP44_Pan_orn																	
Cluster-70816.58017																	
Cluster-70816.150145																	
Cluster-70816.32483																	
CYP4c15_Pan_orn																	
Cluster-70816.25528																	
CYP2L1_Pan_orn																	
ShedC_Pan_orn																	
Cluster-70816.106330																	
ShedB_Pan_orn																	
Cluster-70816.69722																	
CYP11A1a_Pan_orn																	
CYP6a14_Pan_orn																	

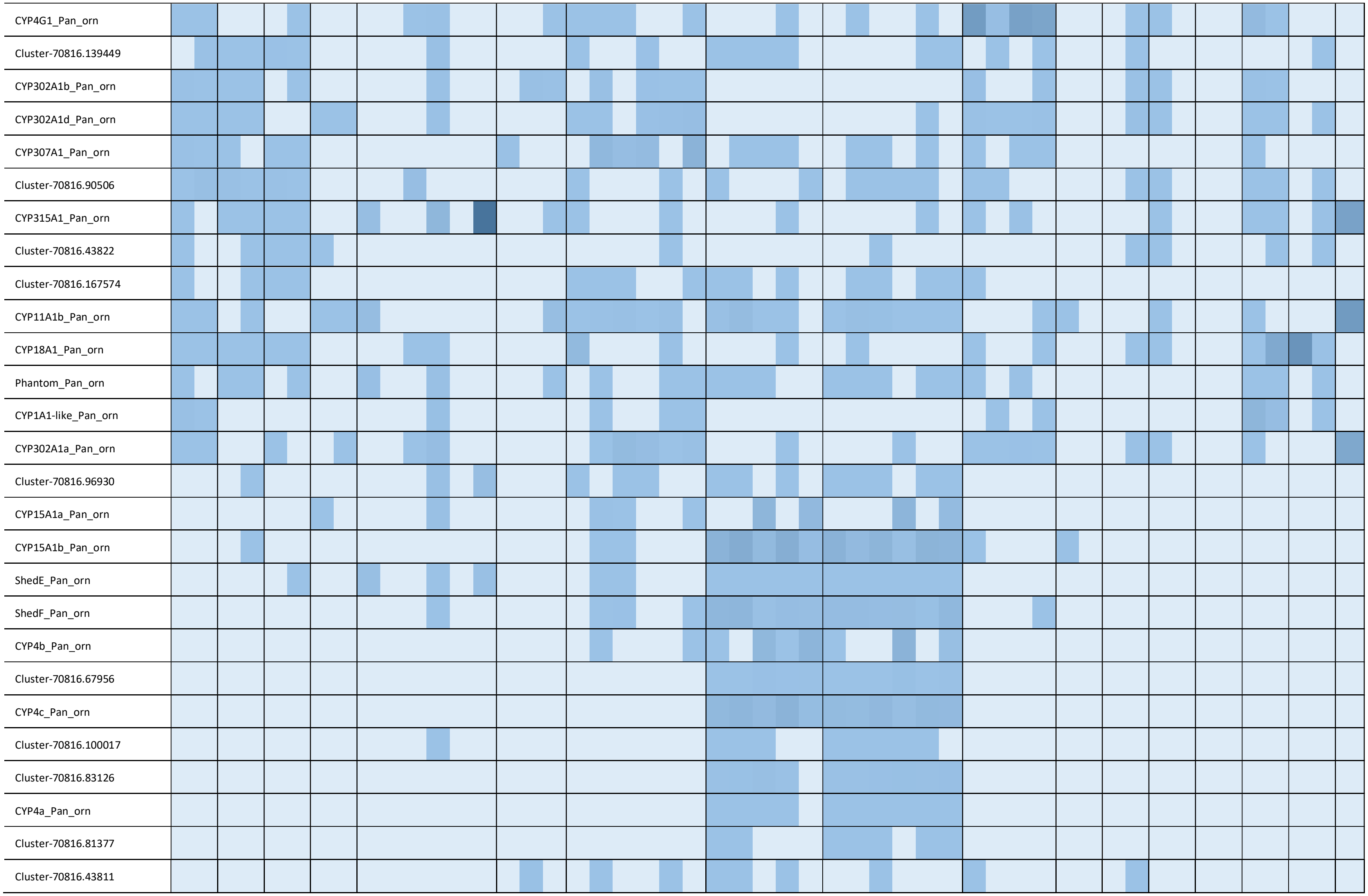
CYP4G1_Pan_orn
Cluster-70816.139449
CYP302A1b_Pan_orn
CYP302A1d_Pan_orn
CYP307A1_Pan_orn
Cluster-70816.90506
CYP315A1_Pan_orn
Cluster-70816.43822
Cluster-70816.167574
CYP11A1b_Pan_orn
CYP18A1_Pan_orn
Phantom_Pan_orn
CYP1A1-like_Pan_orn
CYP302A1a_Pan_orn
Cluster-70816.96930
CYP15A1a_Pan_orn
CYP15A1b_Pan_orn
ShedE_Pan_orn
ShedF_Pan_orn
CYP4b_Pan_orn
Cluster-70816.67956
CYP4c_Pan_orn
Cluster-70816.100017
Cluster-70816.83126
CYP4a_Pan_orn
Cluster-70816.81377
Cluster-70816.43811

CYP302A1c_Pan_orn																	
ShedA_Pan_orn																	
ShedD_Pan_orn																	
Cluster-70816.89805																	
